# Molecular Biology: Getting to the Core of Antimicrobials

**DOI:** 10.1289/ehp.112-1247664

**Published:** 2004-11

**Authors:** Ed Susman

Much research on host defenses against infection has concentrated on the amino acid sequences of antimicrobial peptides in the belief that the order of the acids and their replication reflect how they work against aberrant cells. Now researchers at the University of California, Los Angeles, (UCLA) suggest that the shape the sequences are arranged in may be a critical part of how these peptides work. A new report indicates that host defense systems across the spectrum of life rely on a universal core structure integral to many natural antimicrobial peptides. This core motif may play a key role in preventing or limiting infection, an insight that could accelerate a major advancement in antimicrobial drug development.

“It has been generally accepted that there is a wide diversity in amino acid sequences and sources of antimicrobial peptides,” says study co-investigator Michael Yeaman, a professor of medicine at the David Geffen School of Medicine at UCLA. “But there hasn’t been as much insight into the similarities that might exist that link all of these diverse groups of molecules.”

The gamma (γ)-core motif—so called because it resembles the Greek letter—may be that missing link, providing a key ingredient in the signature of antimicrobial peptides. Yeaman and coauthor Nannette Yount, a molecular biologist at the Los Angeles Biomedical Research Institute, say the γ-core alone can have antimicrobial activity, but also appears to provide a scaffold on which critical modules are configured to create molecules that hunt down microbial pathogens and destroy them in diverse tissue contexts without injury to the host.

The duo studied the amino acid sequences and three-dimensional structures of over 500 antimicrobial peptides, and found the γ-core structure in molecules as diverse as pea defensins, fruit fly drosomycin, pig protegrin, and human hepcidin. Such molecules share the multidimensional signature of antimicrobial peptides. In a paper published 11 May 2004 in *Proceedings of the National Academy of Sciences*, the authors wrote, “This striking multidimensional signature is conserved among disulfide-containing antimicrobial peptides spanning biological kingdoms, and it transcends motifs previously limited to defined peptide subclasses.”

But the sequence, composition, and biochemistry of the amino acids that make up the signature still play a major role, says Yeaman. “We feel that some of the universality identified here may have been missed previously because to identify this signature, we had to look at amino acid sequences in both forward and reverse orientation, and that is not typically done,” he says. “The broad conservation of the multidimensional signature identified may have been missed if we only performed amino acid sequence searches and alignments in a conventional way.”

There are other critical aspects of the γ-core motif as well, Yeaman says. “The amino acid sequence is configured in three-dimensional space so that the γ-core has certain characteristics. For example, electrostatic charge tends to be placed in one part of this motif and hydrophobicity in another; disulfide linkages are also conserved. These hallmark features of the γ-core motif rely on both composition and three-dimensional structure.”

Yeaman and Yount are now translating the motif into peptide mimetics and small molecules, and are designing so-called modular anti-infectives with customized payloads of drugs that attach to the γ-core motif. These compounds are at different stages of development—some are in the design phase, while some have been tested and proven to have antimicrobial efficacy. Still others are being optimized based on data generated in the lab as well as in initial *ex vivo* studies. “We are trying to develop entirely new types of ‘smart’ antibiotics that recognize and act against harmful microbes, particularly those that have become resistant to most all conventional drugs,” Yeaman says.

The work has captured the attention of researchers in the drug development industry. “It’s the structure that defines the signature,” says Steve Projan, vice president of biological technologies at Wyeth Research in Cambridge, Massachusetts, and a consultant to the American Society for Microbiology, based in Washington, D.C. “That structure may be more important than sequence of amino acids. Even if the amino acids are different, it is the overall structure that defines the activity of the molecule.” However, Projan admits, “I’ll be skeptical about the impact of this work until we have a molecule that works by [these] rules and a molecule that also works in an infection model.”

Yeaman suggests that learning how nature has evolved antimicrobial agents may allow scientists to use the γ-core motif or mimetics thereof as the scaffold that will guide the right peptide or molecule to the right target. “Nature has done much of the designing,” he says. “We are capitalizing on the experiments that nature has performed over millions of years [and] trying to integrate the results of that process in new antibiotics.”

## Figures and Tables

**Figure:**
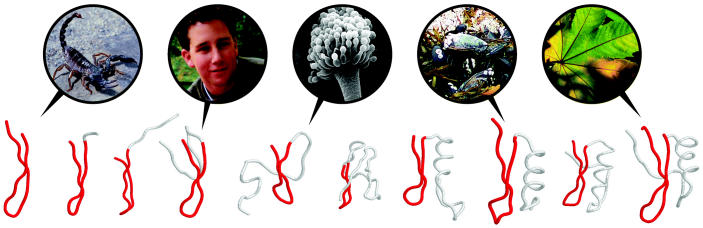
**Common threads.** The γ-core motif, visualized in red above, is seen in antimicrobial peptides from a breadth of organisms, including (left to right) the scorpion, the human, *Aspergillus*, the mussel, and the buckeye tree. The motif appears to provide a scaffold upon which disease-fighting molecules are configured.

